# Diet-microbiota-metabolite interactions in animal health: insights from *Lactobacillus johnsonii*-FM1 and vanillic acid

**DOI:** 10.3389/fvets.2026.1791279

**Published:** 2026-03-19

**Authors:** Dan-Yang Wang, Ling Wang, Lele Fu

**Affiliations:** 1 College of Animal Sciences, Zhejiang University, Hangzhou, China; 2 College of Life Science and Technology, College of Biomedicine and Health, Huazhong Agricultural University, Wuhan, China

**Keywords:** gut microbiota, livestock health, microbial metabolites, microbiota-targeted interventions, prebiotics

In a recent article published in iMetaOmics, Lyu et al. provided compelling evidence that the probiotic strain *Lactobacillus johnsonii*-FM1 (LJ-FM1) exerts potent anti-colorectal cancer effects in murine models (1). This study reveals a novel dual mechanism by which the probiotic LJ-FM1 suppresses colorectal cancer (CRC): on one hand, it reshapes the gut microbiota—enriching beneficial bacteria such as *Bacteroides uniformis* and reducing pathogenic species like *Capnocytophaga canimorsus*; on the other hand, it secretes the key metabolite vanillic acid (VCA). VCA exerts its anti-tumor effects by inhibiting the Wnt/β-catenin signaling pathway, resulting in the downregulation of downstream target genes, including Lgr5 and c-Myc. While this study is firmly rooted in human oncology, its mechanistic insights offer a treasure trove of translational potential for veterinary science, particularly in the field of animal nutrition and metabolism.

Modulating the gut microbiota, reinforcing the epithelial barrier, and producing beneficial metabolites represent not only anti-cancer mechanisms but also fundamental processes for maintaining gut health in livestock and other animals. In intensive animal production systems, disruptions to these very pillars are the root cause of numerous costly and welfare-compromising disorders. For instance, post-weaning diarrhea in piglets is characterized by a dysbiotic shift in the gut microbiota, a breakdown of the intestinal barrier, and a surge in systemic inflammation (2). All those pathophysiological events are strikingly parallel to the tumorigenic environment described by Lyu et al. Similarly, necrotic enteritis in poultry, caused by *Clostridium perfringens* overgrowth, typically arises in the context of dysbiosis and impaired intestinal barrier function (3).

Notably, LJ-FM1 was originally isolated from mice fed black rice (4), underscoring the critical role of diet in shaping a health-promoting microbiota. Similarly, Bachem et al. also found that dietary fiber significantly enriches *Akkermansia muciniphila*, enhancing fiber breakdown and short-chain fatty acid (SCFA) synthesis and ultimately improving melanoma control (5). Together, these findings reinforce a central paradigm: diet-microbiota-metabolite-host interactions form an integrated axis. Therefore, probiotics, prebiotics, and synbiotics can be strategically leveraged to improve animal health by selectively stimulating beneficial microbes or inhibiting pathobionts (6).

However, the same or similar metabolites could exhibit toxic or other adverse effects under different physiological conditions, doses, or in combination with other dietary components. For instance, oligosaccharides like fructooligosaccharides (FOS) and galactooligosaccharides (GOS) are widely regarded as prebiotics that promote the growth of beneficial gut bacteria, support immune function, and may help prevent cancer, cardiovascular disease, and metabolic disorders in humans (7). Yet, paradoxically, both FOS and GOS have been shown to exacerbate clinical symptoms in murine models of colitis (8, 9). Similarly, excessive production of proteolytic metabolites by gut microbes (such as polyamines, biogenic amines, and ammonia), can damage the intestinal epithelial barrier, trigger inflammation, and increase the risk of post-weaning diarrhea in piglets, leading to significant economic losses in livestock production (10). These findings underscore the necessity of adopting a holistic approach that integrates microbiology, metabolomics, and toxicology to fully understand the safety and efficacy profile of any microbiota-targeted strategy before its application in livestock. Thus, although VCA demonstrated anti-inflammatory and anti-proliferative effects in a cancer model, a series of *in vitro* and *in vivo* studies are still required to validate its efficacy and safety in livestock production.

In conclusion, the study by Lyu et al. transcends its original oncological context to provide a robust scientific framework for developing novel strategies to improve animal health. By harnessing specific probiotic strains like LJ-FM1 or designing diets that foster the production of beneficial metabolites like VCA, the animal nutrition community can move beyond simple pathogen suppression toward actively cultivating a resilient and health-promoting gut ecosystem. Such an approach aligns perfectly with the mission of animal nutrition and metabolism to pioneer innovative diagnostics and treatments for nutritional diseases, ultimately enhancing the sustainability and welfare of animal production systems.
